# *Comamonas kerstersii* bacteremia in a patient with acute perforated appendicitis: A rare case report

**DOI:** 10.1097/MD.0000000000009296

**Published:** 2018-03-30

**Authors:** Yun-heng Zhou, Hong-xia Ma, Zhao-yang Dong, Mei-hua Shen

**Affiliations:** a Department of Clinical Laboratory, Shanghai Provincial Crops Hospital, Chinese People's Armed Police Forces; b Department of Physical Examination Center, Shanghai East Hospital, Tongji University; c Department of Burns and Wound Repair Surgery; d Department of Emergency, Shanghai Provincial Crops Hospital, Chinese People's Armed Police Forces, Shanghai, China.

**Keywords:** bacteremia, *Comamonas kerstersii*, perforated appendicitis

## Abstract

**Rationale:**

Comamonas species are rarely associated with human infections. Recent reports found that *Comamonas kerstersii* was associated with severe diseases such as abdominal infection and bacteremia. However, *C. kerstersii* maybe be confused with *Comamonas testosteroni* using the automatic bacterial identification systems currently available.

**Patient concerns:**

A 31-year-old man who had onset of left upper abdominal pain developed clinical manifestations of right lower abdominal pain and classic migration of pain at the temperature of 39°C. The positive strain of aerobic and anaerobic bottles of blood cultures was identified.

**Diagnoses:**

The patient was diagnosed as acute peritonitis and perforated appendix with abdominal abscess.

**Interventions:**

The bacterium was identified by routine methods, MALDI-TOF-MS and PCR amplification of the 16S rRNA. The patient was treated with exploratory laparotomy, appendectomy, tube drainage, and prescribing antibiotic treatment.

**Outcomes:**

The patients were discharged with complete recovery. The organisms were confirmed as *C*. *kerstersii* by MALDI-TOF-MS and a combination of the other results.

**Lessons:**

Our findings suggest that *C*. *kerstersii* infection occurs most often in association with perforated appendix and bacteremia. We presume that *C*. *kerstersii* is an opportunistic pathogen or commensal with the digestive tract and appendix bacteria.

## 1 Introduction

The genus *Comamonas* was originally created in 1985, and it included a single species, *Comamonas terrigena (C. terrigena)*.^[1]^ In 1987, *Comamonas testosteroni* and *Comamonas acidovorans* were reclassified as members of the *Comamonas* genus. *C*. *acidovorans* was subsequently reclassified as *Delftia acidovorans* on the basis of its 16S rRNA gene sequence in 1999.^[2]^*Comamonas kerstersii (C*. *kerstersii)* was described as 1 of 3 genotypically separate groups of *C. terrigena* in 2003.^[3]^ Now, *Comamonas* genus contains 17 species including *C. terrigena,C. aquatica*, *C. kerstersii, C*. *testosteron*i, *C*. *denitrificans*, *C*. *nitrativorans*, *C*. *koreensis* and others. *Comamonas* species have a wide geographic distribution and are commonly found in soil, plants, animal, water saprophytes, and in humidifier reservoir water.^[4]^

*Comamonas* species are rarely associated with human infections.^[5]^ However, in recent years, several publications have incriminated *C. testosteroni* and *C*. *kerstersii* in human diseases, including severe invasive infections, such as abdominal infection and bacteremia.^[6–14]^ However, *C*. *kerstersii* maybe be confused with C. *testosteroni* because of the difficulties in accurately identifying it using the automatic bacterial identification systems currently available. Some important biochemical tests, matrix-assisted laser desorption ionization–time of flight mass spectrometry (MALDI-TOF-MS) and gene sequencing by polymerase chain reaction (PCR) amplification of the 16S rRNA can confirm the specific *Comamonas* species. To the best of our knowledge, this is the first report of *C*. *kerstersii* bacteremia in a patient with acute perforated appendicitis.

## 2 Case presentation

A 31-year-old man presented to the emergency department of our hospital with onset of left upper abdominal pain followed by nausea and vomiting at a temperature of 37.5°C. His white blood cell was 11.6 × 10^9^/L, and differential white blood count were: neutrophils 59.4%, lymphocytes 29.2%, monocytes 7.2%, eosinophils 4.1%. Serum C-reactive protein level was 23.5 mg/L. Elevated alanine transaminase level (101 U/L) was found. Other laboratory data were within the normal range. His chest and abdominal X-ray and ultrasonography were normal. The patient was treated with cefuroxime axetil according to empirical antibiotic routines. The next day, he had a temperature of 39°C with right lower abdominal pain and classic migration of pain. Aerobic and anaerobic bottles of blood cultures were drawn and incubated at 35°C; However, he refused to accept surgical intervention due to personal reason. A follow-up visit revealed that he was diagnosed with acute peritonitis and perforated appendix with abdominal abscess. He was discharged with complete recovery after exploratory laparotomy, appendectomy, tube drainage, prescribing antibiotic treatment for 14 days (Cefuroxime and metronidazole) and an observation period of 6 days.

As soon as the aerobic blood culture bottle became positive 18 hours after sampling and the anaerobic blood culture bottle became positive 20 hours after sample collection, they were transferred into Colombia blood agar, macconkey, nutrition agar and chocolate agar. After 24 hours of incubation at 35°C in ambient air, growth of a nonfermenting Gram-negative bacillus was observed. Other tests showed that oxidase and catalase activities were positive. 4-hour rapid urea hydrolysis was negative. Strains also grew on the 4 types of agar at 30°C and 42°C. The strain was initially identified as *Bordetella bronchiseptica* by SIEMENS MicroScan walkaway 96 plus system (Siemens, New York, NY). The minimum inhibitory concentration results showed that all the antibiotics except ciprofloxacin [(minimum inhibitory concentration, MIC) S≤1, R≥4], levofloxacin(MIC S≤2, R≥8) and trimethoprim-sulfamethoxazole (MIC S≤2/38, R≥4/36) were sensitive. The isolates were identified as *C*. *testosteroni* by VITEK 2, BD phoenix100 and ATB expression systems at different hospitals in Shanghai.

Identification was also carried by MALDI-TOF-MS. The spectral score for *C*. *kerstersii* was 1.815, followed by *Comamonas aquatic* of the score 1.673. Because the score was less than 2.0, we proceeded to extract DNA and performed PCR amplification of the 16s rRNA gene sequencing for bacterial identification, which showed it was closest with *Comamonas kerstersii* strain 8943 sequence (CP020121.1, complete genome, Max 2534, Total score 12673, Query cover 100%, Identity 99%) and *C. kerstersii* strain LMG 5323 sequence (AJ430348.1, partial 16S rRNA gene, Max 2525, Total score 2525, Query cover 100%, Identity 99%). Considered that the biochemical tests, the organisms were confirmed as *C*. *kerstersii* by MALDI-TOF-MS and a combination of the other results.

## 3 Discussion

Since 1987,^[6–11]^ 34 patients infected with *C. testosteroni* around the world have been reported: 16 with bloodstream infections, 10 with abdominal cavity infections, 8 with other kinds of infections. Among these, Gul et al were the first to report *C. testosteroni* from the blood cultures of a 22-year-old man with a perforated appendix in Turkey, and the organism was identified by Mini API to be sensitive to all antibiotics tested.^[9]^ Tsui et al presented 2 strains from bacteremia identified by the Phoenix100 System in 2011: a 54-year-old alcoholic patient with left leg cellulitis and a 73-year-old male with chronic hepatitis B infection, liver cirrhosis, and hepatocellular carcinoma after transarterial embolization. The 2 strains were sensitive to a broad range of antibiotics, including all tested cephalosporins and quinolones.^[12]^ Opota et al also commented that there were 32 *Comamonas* sp. strains and 38 *D*. *acidovorans* strains isolated from 1997 to 2013 in his hospital, which were isolated primarily from respiratory tract samples (33%), urogenital tract samples (23%), and digestive tract samples (21%), while bacteremia represented 5% (3 patients) of the cases.^[13]^

However, it is possible that some of the isolates identified as *C. testosteroni* might have been *C. kerstersii,* as *C. kerstersii* is not found in the VITEK, ATB, API, Siemens, and BD system databases, in which there are only 2 types of *Comamonadaceae*: *D. acidovorans* and *C. testosteroni*. In the present case, the organisms were initially identified as *B. bronchiseptica,* which was obviously not correct because it is a strict aerobe that grows slowly and forms small colonies in 48 hours.^[14]^

To date, there are only 8 cases of *C. kerstersii* reported in the literature,^[13,15,16]^ therefore, our patient represents the ninth reported case of *C. kerstersii* infection (Table 1). All of the *C*. *kerstersii* isolates were identified by MALDI-TOF-MS, which is a rapid and accurate method to differentiate between *Comamonas* species. Some tests can also differentiate *C. kerstersii* from other *Comamonas* species according to schemes proposed by Wauters et al,^[17]^ such as sensitivity to colistin and deferoxamine, nonuse of testosterone, a negative pyrrolidone arylamidase test, growth at 42°C, and a positive tyrosine hydrolysis.

**Table 1:**
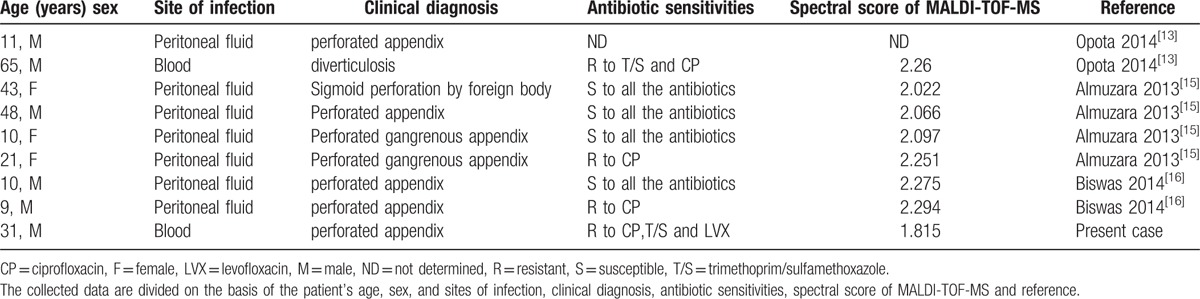
Clinical and microbiological characteristics of the 9 cases of *C. kerstersii* infections.

Drug sensitivity tests showed that the isolates were sensitive to a broad range of antibiotics. Among the 9 cases, 2 were identified in bacteremia patients with diverticulosis and perforated appendixes and the predominant source of infection were in the peritoneal fluid of the abdominal cavity(7/9). The main clinical diagnosis of these patients is perforated appendix(7/9), followed by sigmoid perforation and diverticulosis, which demonstrates the association of *C. kerstersii* with severe diseases. Aside from the previously reported cases of *C. testosteroni* infections, Opota et al^[13]^ reported the first *C*. *kerstersii* bloodstream infection in a patient with diverticulosis. The present case is the first report of *C. kerstersii* bacteremia in a patient with acute perforated appendicitis. *Comamonas* species infection has been associated with exposure to contaminated fish tank water or exploration of the abdominal cavity.^[18]^ Thus, we presume that *C*. *kerstersii* is an opportunistic pathogen or commensal with the digestive tract and appendix bacteria.

## 4 Conclusion

In summary, *Comamonas kerstersii* infection occurs most often in association with severe diseases, such as perforated appendix and bacteremia. This strain is always sensitive to a broad range of antibiotics. However, *C*. *kerstersii* is easily confused with C. *testosteroni* by automatic bacterial identification systems currently available on the market. Overall, MALDI-TOF-MS and gene sequencing are a more accurate approach to identify the species than others. Further research is required to clarify the origins of this organism.

## Author contributions

**Data curation:** Y.H. Zhou.

**Formal analysis:** H.X. Ma.

**Formal analysis:** Y.H. Zhou.

**Funding acquisition:** M.H. Shen.

**Methodology:** H.X. Ma.

**Resources:** M.H. Shen.

**Supervision:** Z.Y. Dong.

**Writing – original draft:** Y.H. Zhou.

**Writing – review & editing:** Y.H. Zhou.

## Acknowledgments

We are grateful to all of the contributors of the departments of laboratory medicine in different hospitals for their identification of this strain. This work was supported by the Key Programs of Science and Technology Commission Foundation of Changning District, Shanghai (CNKW2016Z05) and the National Nature Science Foundation of China (No. 81401855).

## References

[1] De Vos P Kersters K Falsen E *Comamonas* Davis and Park 1962 gen. nov., nom. rev. emend., and *Comamonas terrigena* Hugh 1962 sp. nov. Int J Syst Bacteriol 1985;35:443–53.

[2] Wen A Fegan M Hayward C Phylogenetic relationships among members of the Comamonadaceae and description of Delftia acidovorans (den Dooren de Jong 1926 and Tamaoka et al 1987) gen. nov., comb. nov. Int J Syst Bacteriol 1999;49:567–76. 10319477 10.1099/00207713-49-2-567

[3] Wauters G De Baere T Willems A Description of Comamonas aquatica comb. nov. and *Comamonas kerstersii* sp. nov. for two subgroups of *Comamonas terrigena* and emended description of *Comamonas terrigena*. Int J Syst Evol Microbiol 2003;53:859–62. 12807213 10.1099/ijs.0.02450-0

[4] Nakipoglu Y Erturan Z Buyukbaba-Boral O Evaluation of the contaminant organisms of humidifier reservoir water and investigation of the source of contamination in a university hospital in Turkey. Am J Infect Control 2005;33:62–3. 15685139 10.1016/j.ajic.2004.09.007

[5] Koneman EW Allen SD Janda WM Color atlas and Testbook of Diagnostic Microbiology. 1997;New York: J. B. Lippincott Company, 264–77.

[6] Abraham JM Simon GL *Comamonas testosteroni* bacteremia: a case report and review of the literature. Infect Dis Clin Pract 2007;15:272–3.

[7] Farshad S Norouzi F Aminshahidi M Two cases of bacteremia due to an unusual pathogen, *Comamonas testosteroni* in Iran and a review literature. J Infect Dev Ctries 2012;6:521–5. 22706196 10.3855/jidc.2215

[8] Bayhan Gi Tanir G Karaman I *Comamonas testosteroni*: an unusual bacteria associated with acute appendicitis. Balkan Med J 2013;30:447–8. 25207159 10.5152/balkanmedj.2013.9135 PMC4115943

[9] Gul M Ciragil P Bulbuloglu E *Comamonas testosteroni* bacteremia in a patient with perforated acute appendicitis. Short communication. Acta Microbiol Immunol Hung 2007;54:317–21. 17896478 10.1556/AMicr.54.2007.3.6

[10] Nseir W Khateeb J Awawdeh M Catheter-related bacteremia caused by *Comamonas testosteroni* in a hemodialysis patient. Hemodial Int 2011;15:293–6. 21223488 10.1111/j.1542-4758.2010.00524.x

[11] Barbaro DJ Mackowiak PA Barth SS *Pseudomonas testosteroni* infections: eighteen recent cases and a review of the literature. Rev Infect Dis 1987;9:124–9. 3823716 10.1093/clinids/9.1.124

[12] Tsui TL Tsao SM Liu KS *Comamonas testosteroni* infection in Taiwan:reported two cases and literature review. J Microbiol Immunol Infect 2011;44:67–71. 21531356 10.1016/j.jmii.2011.01.013

[13] Opota O Ney B Zanetti G Bacteremia caused by *Comamonas kerstersii* in a patient with diverticulosis. J Clin Microbiol 2014;52:1009–12. 24371242 10.1128/JCM.02942-13 PMC3957752

[14] Choy KW Wulffraat NM Wolfs TF *Bordetella bronchiseptica* respiratory infection in a child after bone marrow transplantation. Pediatr Infect Dis J 1999;18:481–3. 10353531 10.1097/00006454-199905000-00022

[15] Almuzara MN Cittadini R Vera Ocampo C Intra-abdominal infections due to *Comamonas kerstersii*. J Clin Microbiol 2013;51:1998–2000. 23576541 10.1128/JCM.00659-13 PMC3716083

[16] Biswas JS Fitchett J O’Hara G *Comamonas kerstersii* and the perforated appendix. J Clin Microbiol 2014;52:3134. 24829228 10.1128/JCM.00909-14 PMC4136147

[17] Wauters G Vaneechoutte M Versalovic J Carroll KC Approaches to the identification of aerobic Gram-negative bacteria. Manual of Clinical Microbiology. Washington, DC: ASM Press; 2011 539–58.

[18] Smith MD Gradon JD Bacteremia due to Comamonas species possibly associated with exposure to tropical fish. South Med J 2003;96:815–7. 14515927 10.1097/01.SMJ.0000051869.86765.D9

